# Senolytics: charting a new course or enhancing existing anti-tumor therapies?

**DOI:** 10.1007/s13402-024-01018-5

**Published:** 2024-12-04

**Authors:** Konrad Czajkowski, Mariola Herbet, Marek Murias, Iwona Piątkowska-Chmiel

**Affiliations:** 1 Department of Toxicology, Faculty of Pharmacy, Medical University of Lublin, Lublin, Poland; 2 Department of Toxicology, Poznan University of Medical Sciences, Poznań, Poland

**Keywords:** Cell senescence, Cancer, Senotherapy, SASP, One-Two Punch therapy

## Abstract

Cell senescence is a natural response within our organisms. Initially, it was considered an effective anti-tumor mechanism. However, it is now believed that while cell senescence initially acts as a robust barrier against tumor initiation, the subsequent accumulation of senescent cells can paradoxically promote cancer recurrence and cause damage to neighboring tissues. This intricate balance between cell proliferation and senescence plays a pivotal role in maintaining tissue homeostasis. Moreover, senescence cells secrete many bioactive molecules collectively termed the senescence-associated secretory phenotype (SASP), which can induce chronic inflammation, alter tissue architecture, and promote tumorigenesis through paracrine signaling. Among the myriads of compounds, senotherapeutic drugs have emerged as exceptionally promising candidates in anticancer treatment. Their ability to selectively target senescent cells while sparing healthy tissues represents a paradigm shift in therapeutic intervention, offering new avenues for personalized oncology medicine. Senolytics have introduced new therapeutic possibilities by enabling the targeted removal of senescent cells. As standalone agents, they can clear tumor cells in a senescent state and, when combined with chemo- or radiotherapy, eliminate residual senescent cancer cells after treatment. This dual approach allows for the intentional use of lower-dose therapies or the removal of unintended senescent cells post-treatment. Additionally, by targeting non-cancerous senescent cells, senolytics may help reduce tumor formation risk, limit recurrence, and slow disease progression. This article examines the mechanisms of cellular senescence, its role in cancer treatment, and the importance of senotherapy, with particular attention to the therapeutic potential of senolytic drugs.

**Graphical abstract:**

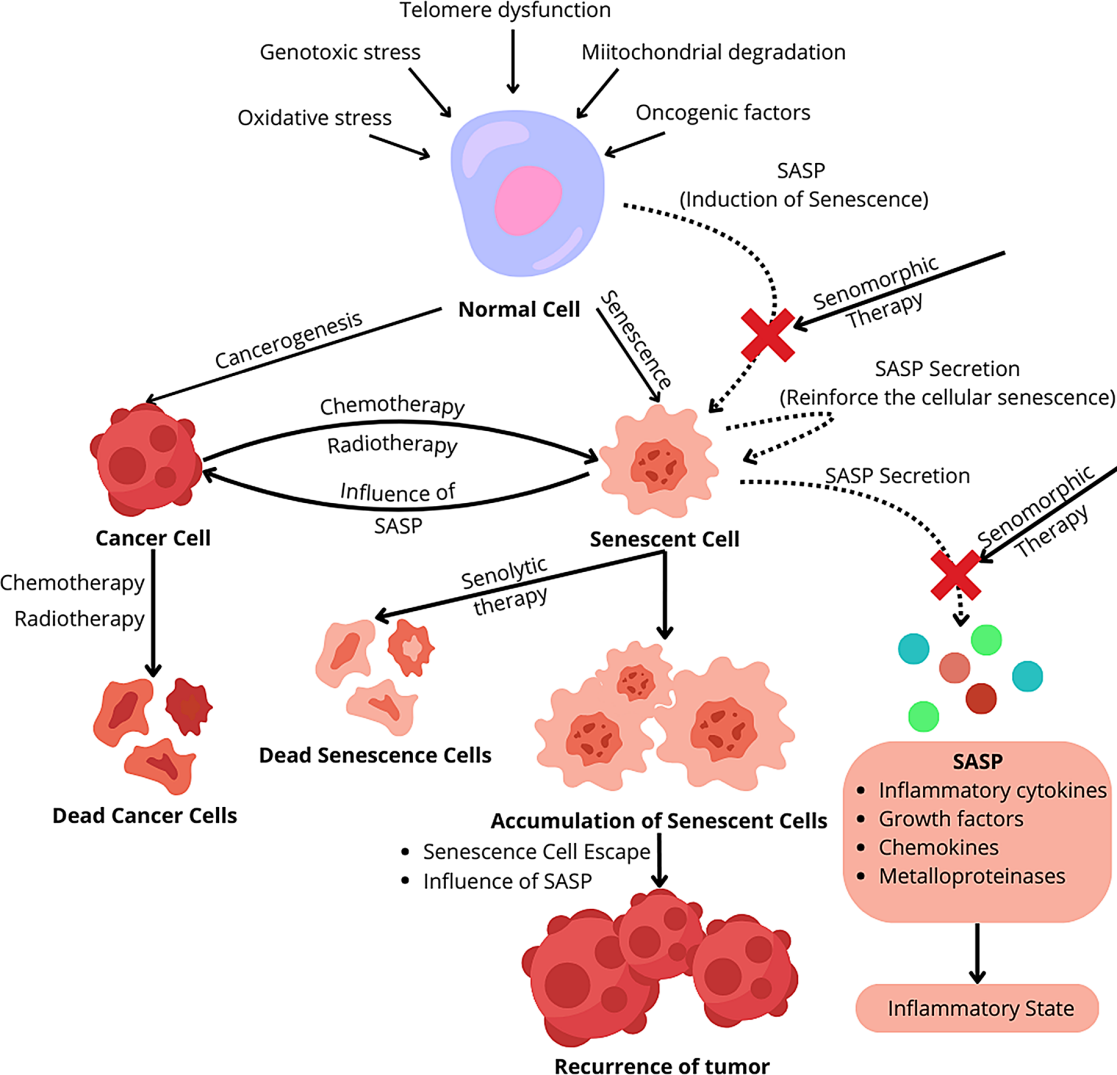

## Introduction

Cancer persists as one of the foremost health challenges in the contemporary world. In 2019, cancer was responsible for the deaths of over ten million people, making them the second leading cause of death after cardiovascular diseases [1]. The timely diagnosis of patients, coupled with intensive therapies that impose a considerable burden on the body [2], and the acceleration of aging in society [3], wherein the elderly demonstrate a heightened incidence of all cancer forms and the highest mortality rate [1], underscores the imperative for scientists to pursue novel, more efficacious, and less taxing therapies. Cellular senescence is a natural defense mechanism in our bodies [4]. Upon oncogenic activation, the body triggers cell senescence, inhibiting their proliferation, even at the pre-cancerous stage [4]. This mechanism is also evident in currently employed anticancer therapies [5]. Therapies like chemotherapy and radiotherapy, designed to induce extensive DNA damage and subsequently eliminate rapidly dividing cancer cells, have been found to provoke a robust senescence reaction [6]. Presently developed pro-senescence therapies concentrate on the deliberate induction of cellular senescence [7]. Research on these therapies points to a positive impact of senescence induction, particularly in the initial stages of the disease [8]. Unfortunately, it has been observed that the prolonged presence of senescent cancer cells could lead to the emergence of new cancers, metastases, and recurrences [6]. For this reason, many scientists have redirected their focus towards therapies based on the opposite mechanism. Senotherapy, in contrast to conventional anti-cancer therapies (chemotherapy and radiotherapy), aims to inhibit the senescence process [9]. The selective elimination of senescent cells or support for the body in this process through the modulation of autophagy in them, coupled with limiting the impact of senescent cells on their environment, may prove highly effective in anti-cancer therapy [9].

In this review, our focus would be on assessing the impact of cellular senescence on cancer development, evaluating current therapies, and analyzing the potential of senotherapy in the context of individual and synergistic cancer treatment.

## Cellular senescence

Over 60 years ago, L. Hyflick, in his pioneering work, first used the term “cellular senescence” [10]. He defined it as a permanent exit from the cell cycle caused by the limited proliferative capacity of cultured human fibroblasts [10]. Currently, cellular senescence is understood as the response of cells to stress induced by both endogenous and exogenous factors, such as oncogenic factors, and genotoxic stress, telomere dysfunction, or mitochondrial degradation [11]. Thanks to numerous studies conducted in recent decades, we are aware that cellular senescence plays a significant role in the functioning of our organism [12–15]. Its impact is not limited to the aging process of the organism [12] but also extends to participation in wound healing [13], embryonic development [14], and tumor suppression [15].

### Features of cellular senescence

The phenomenon of cellular senescence is a complex and multi-stage process, the course, and characteristics of which are often conditioned by numerous factors, such as the origin of the stress factor or the specificity of cells undergoing senescence [16, 17]. Despite the complexity of cellular senescence, several hallmark features can suggest that cells have entered this state. The most visually apparent sign is a change in cell size and shape. Due to activation of the mTOR pathway, which regulates cell growth [18], and modulation of the ATF6α pathway, affecting cytoskeletal structure [19], senescent cells often take on an enlarged and irregular morphology [18, 19]. Additionally, increased cellular granularity, likely due to mTOR activation, is commonly observed [18]. Beyond these structural changes, senescent cells also exhibit cell cycle arrest, remaining mostly in the G1 phase [20]. Current research indicates that the cell cycle arrest depends on the activity of cyclin-dependent kinase inhibitors (CDK) p16 and p21, often regulated by the main tumor suppressor protein p53, whose elevated level is frequently used as a marker of senescence detection [21]. Furthermore, senescent cells exhibit resistance to apoptosis [22]. This resistance is partially due to heightened and sustained activity of the transcription factor cAMP response element-binding protein (CREB), which prevents inhibition of the anti-apoptotic protein BCL-2 [22]. The level of BCL-2 protein may thus serve as an additional marker of senescence. In senescent cells, alterations in plasma membrane composition can also occur [23]. Research by Althubiti et al. showed that in senescent bladder cancer cells of the Ejp21 and Ejp16 lines, 107 and 132 unique plasma membrane proteins, respectively, were detected - proteins absent in non-senescent cancer cells [23]. However, only 17 of these proteins were shared across both cell lines, suggesting high variability in this process and indicating the limited potential of many of these proteins as universal markers of senescence [23]. Among plasma membrane proteins, DPP4 (dipeptidyl peptidase-4) is considered a key protein in the context of cellular senescence [24]. It has been shown not only that DPP4 expression increases in senescent cells but also that modulation of this protein’s expression can either induce or counteract cellular senescence [24]. Additionally, as a surface protein, DPP4 serves as a promising target for DPP4-specific antibodies, enabling selective elimination of senescent cells [24]. Senescent cells are also characterized by reduced mitophagy, leading to the accumulation of dysfunctional mitochondria and a noticeable increase in mitochondrial presence within the cell, which may serve as another indicator of cellular senescence [25]. Likewise, the number of lysosomes within the cell increases [26]. As lysosome quantity rises, so does their activity, manifesting as elevated levels of the lysosomal enzyme Senescence-Associated β-Galactosidase (SA-β-gal) [26]. Currently, SA-β-gal activity is considered one of the most crucial markers of cellular senescence [26]. Unfortunately, the increased activity of SA-β-gal is not a specific marker for senescent cells, as its elevation can also be observed in certain conditions in hair follicles, sebaceous glands, and activated macrophages [27, 28]. In senescent cells, the nuclear membrane can undergo structural remodeling, leading to its destabilization [29]. This occurs partly due to a significant reduction in the expression of LaminB1, a key protein component of the nuclear membrane [29]. Additional changes can also arise within the nucleus. One characteristic alteration in senescent cells is the formation of senescence-associated heterochromatin foci (SAHF) [30]. Researchers have shown that SAHF suppresses the expression of proliferation-promoting genes and dampens DNA damage signaling, potentially contributing to cell cycle arrest while preventing apoptosis [30]. A key factor in driving cells into senescence is the accumulation of significant DNA damage [18]. Among such damages are double-strand breaks (DSB), which can activate a DNA damage response (DDR) [18]. DDR signaling involves the release of signaling and repair proteins, such as γ-H2AX and phosphorylated p53, which accumulate at DSB sites to form DNA damage foci [18]. When DNA damage is reparable, these DNA damage foci are temporary; however, in cases of severe damage, such as complex DNA breaks, these foci become persistent [18]. Persistent DNA damage leads to the formation of chromatin-modifying segments known as DNA segments with chromatin alterations reinforcing senescence (DNA-SCARS) [18]. Both transient DNA damage foci and DNA-SCARS are detectable, making proteins like γ-H2AX and phosphorylated p53 reliable markers of senescence [18]. Additionally, senescent cells secrete a range of chemokines, cytokines, and growth factors, collectively known as the senescence-associated secretory phenotype (SASP) [31]. Since SASP plays a crucial role in the cellular senescence process, its levels and precise composition can serve as valuable markers of senescence [31].

#### Senescence-associated secretory phenotype—SASP

The senescence-associated secretory phenotype, also known as the senescence-messaging secretome, functions as a collection of proteins released by senescence cells into their microenvironment, serving as a key marker of cellular aging [32, 33]. Its composition includes proteins such as inflammatory cytokines, growth factors, chemokines, and metalloproteinases [34]. Diverse functions and actions of SASP factors have been reported [33]. In an autocrine mechanism, SASP factors reinforce the cellular senescence of the senescent cells themselves, while in a paracrine mechanism, they are responsible for inducing senescence in surrounding cells [35]. A significant physiological function of SASP is its involvement in the repair of damaged tissues [13, 36]. Studies have demonstrated that SASP factors recruit immune cells to the site of tissue damage, stimulating the removal of damaged and aging cells [13, 36]. Additionally, among SASP factors, there are growth factors such as vascular endothelial growth factor (VEGF), platelet-derived growth factor (PDGF), and insulin-like growth factor 1 (IGF-1), which, when secreted into the environment by senescence cells, stimulate the repair and regeneration of surrounding tissues [13]. Numerous studies have indicated that SASP does not exert solely positive effects [31, 34, 37, 38]. Senescent cells, through the secretion of SASP factors into their environment, influence neighboring non-senescent cells and the extracellular matrix (ECM), leading to inflammation, fibrosis, and programmed death of healthy cells, while simultaneously becoming resistant to apoptosis [34].

The impact of SASP on cancer cells is also ambiguous. Numerous studies indicate that, depending on various factors, SASP can either promote or suppress tumor development. Ruscetti et al. demonstrated that senescent pancreatic ductal adenocarcinoma (PDAC) cells in mice activated endothelial cells through the release of SASP factors into the tumor microenvironment [39]. This, in turn, led to an accumulation of CD8+ T immune cells, contributing to tumor reduction [39]. The role of immune system activation as an anti-tumor mechanism of the SASP is also highlighted by Iannello et al. [40]. Their research showed that senescent liver cancer cells in mice secreted SASP factors, including the chemokine CCL2, which triggered localized inflammation within the tumor [40]. This led to the accumulation of NK cells, effectively contributing to tumor elimination [40]. However, other studies have shown contrasting effects [41]. For instance, senescent hepatocytes located in the peritumoral region suppressed the immune response, thereby promoting the development of hepatocellular carcinoma (HCC) [41]. By releasing chemokines into their surroundings, senescent hepatocytes caused an accumulation of immature myeloid cells, leading to the inhibition of NK cell function and a weakened immune response against cancer cells [41]. SASP induction by senescent cancer cells can also improve tumor vascularization, a phenomenon that may be considered beneficial, as demonstrated in previous studies on PDAC cells [39]. Researchers noted that the SASP factors secreted by senescent PDAC cells included pro-angiogenic agents that contributed to increased tumor vascularization [39]. This, in turn, could potentially improve cytotoxic drug availability and enhance therapeutic outcomes [39]. However, despite evidence suggesting the positive effects of improved tumor vascularization, there is also a risk of enhanced cancer cell activity and accelerated tumor growth [42]. Moreover, SASP factors may contribute to the increased migratory capabilities of cancer cells. By secreting cytokines such as IL-6 and IL-8, senescent cancer cells can induce epithelial-mesenchymal transition (EMT), leading to a more invasive phenotype and increasing the risk of metastasis [33, 43].

### Senescence cell escape

For many years, senescence has been considered an irreversible form of growth arrest [44]. However, in recent years, numerous studies have suggested that certain subpopulations of senescent cancer cells may regain the ability to proliferate by escaping the senescence process [45, 46]. Senescent cancer cells can temporarily enter a dormant state, rendering them insensitive to conventional treatments [47]. Subsequently, under appropriate conditions, they may re-enter the cell cycle, leading to disease recurrence or even further progression [47].

There is no uniform mechanism for escaping senescence [48]. In one study, the potentially significant role of glutamine in this phenomenon was observed [49]. Deficiency of this substance inhibited senescence escape in the majority of examined cell lines [49]. Among the cells that successfully escaped senescence, increased expression of the SLC1A5 variant of mitochondrial glutamine transporter and enhanced glutamine synthesis were noted, safeguarding these cells against glutamine deprivation [49]. Glutamine, replacing glucose as the substrate for ATP production, plays a crucial role as a vital nutrient for cancer cells [50]. The heightened expression of the mitochondrial glutamine transporter, SLC1A5, results in an increased abundance of this transporter in the mitochondrial membrane [50]. This, in turn, facilitates the augmented transport of glutamine into the mitochondria of cancer cells, ultimately leading to heightened ATP production induced by glutamine and the synthesis of glutathione [50]. Consequently, cancer cells can generate energy under anaerobic conditions and sustain their capacity for cell growth [50]. Furthermore, it has been demonstrated that the increased production of glutathione, which regulates the level of reactive oxygen species (ROS) in cancer cells, contributes to the resistance of pancreatic cancer cells to gemcitabine [50]. The effectiveness of gemcitabine is closely tied to the level of ROS [50]. Other studies emphasize the crucial role of the p21 protein in maintaining the senescence phenotype [50]. Inactivation of this protein in senescent colorectal cancer cells led to the re-expression of cell cycle regulators such as polo-like kinase 1 (PLK1) and cell division cycle 25 (CDC25) [51]. This resulted in the restoration of proliferative capacity and escape from senescence [51].

Many mechanisms of senescence escape remain unknown. The mechanism often utilized by senescent cells depends on numerous factors, such as cell type or the method of senescence induction [48]. Furthermore, despite the knowledge regarding the occurrence of some of these mechanisms, we still cannot explain the causal relationship between the observed changes and senescence escape for each of them [47].

## Therapy-induced senescence

Conventional approaches to cancer treatment, such as chemotherapy or radiotherapy, involve the use of cytostatic drugs or ionizing radiation to eradicate cancer cells [52]. Despite these therapies not being initially designed to trigger cellular senescence, it was discovered as early as 1998 that cisplatin could induce cellular senescence in head and neck cancer cells [53]. Presently, our understanding allows us to assert that the predominant methods of radiotherapy and cytostatic drugs may induce cellular senescence [54]. Moreover, recent studies suggest that the induction of cellular senescence, rather than the promotion of apoptosis through radiotherapy or chemotherapy, might be the main mechanism for impairing the self-renewal capacity of cancer cells, particularly in the context of solid tumors [55]. The phenomenon of cellular senescence in cancer cells triggered by radiotherapy or chemotherapy is termed therapy-induced senescence (TIS) [54].

### The impact of chemotherapy on the aging of cancer cells

**Fig. 1:**
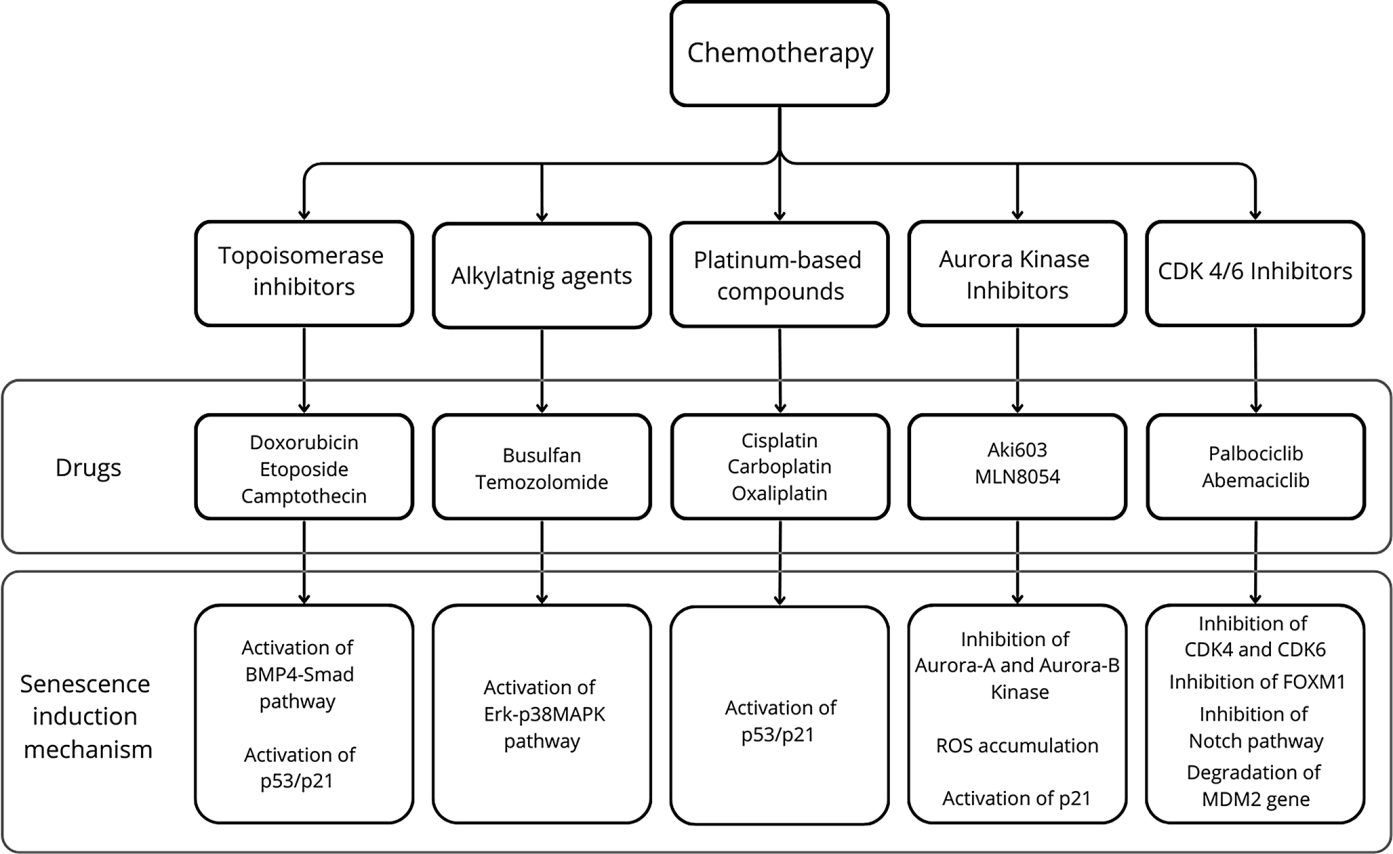
Presentation of the mechanisms of senescence induction by chemotherapy. The five main groups of chemotherapeutic drugs capable of inducing senescence are Topoisomerase inhibitors, Alkylating agents, and Platinum-based compounds, Aurora Kinase inhibitors and CDK4/6 inhibitors. Topoisomerase inhibitors such as Doxorubicin, etoposide, and camptothecin can induce senescence by activating the BMP-4 Smad pathway and activating p53, p21^Cip1,^ and p16^INK4^. Alkylating agents such as busulfan and temozolomide can induce senescence by activating the Erk-p38MAPK pathway. Platinum-based compounds such as cisplatin, carboplatin, and oxaliplatin can induce senescence by activating p53/p21 and p16. Aurora kinase inhibitors, such as Aki603 and MLN8054, can induce senescence by inhibiting Aurora-A and Aurora-B kinases, which results in the accumulation of ROS and activation of p21. CDK4/6 inhibitors such as palbociclib and abemaciclib can induce senescence by inhibiting CDK4, CDK6, FOXM1, Notch pathway and degradation of MDM2 gene

Chemotherapy involves the use of cytotoxic substances to cause damage to rapidly dividing cancer cells. These cytotoxic agents, commonly referred to as chemotherapeutic drugs, can act in several ways, such as interfering with DNA replication, disrupting cell division, or triggering apoptosis (programmed cell death) [56]. The primary goal of chemotherapy is to target and eliminate cancer cells throughout the body, thereby inhibiting their ability to grow and divide [57]. By targeting rapidly dividing cells, chemotherapy aims to eliminate not only the primary tumor but also any potential cancer cells that may have spread to other parts of the body. Diverse studies unequivocally indicate that chemotherapeutics also could induce the senescence process in both cancerous and healthy cells [56, 58–60] (Fig. 1).

#### Topoisomerase inhibitors

Topoisomerase inhibitors are a class of anticancer drugs whose mechanism of action is based on blocking topoisomerase enzymes from re-ligating the DNA strands after supercoiling, thus preventing DNA replication [61]. The most frequently used drug from this group is doxorubicin, which is used in the treatment of cancers located in the lungs and breasts, as well as in the treatment of lymphomas and acute lymphocytic leukemia [61]. Doxorubicin has been reported to induce significant senescence in breast cancer cell lines, particularly promoting SA-β-gal expression in MCF7 cells line indicating a substantial impact of p53 on senescence induction [58]. Additionally, a considerable influence of BMP4-Smad pathway activation and central cell cycle inhibitors: p21^Cip1^ and p16^INK4^, on cellular aging induction by this drug has been demonstrated [59]. Studies also reveal that doxorubicin enhances the expression of SASP, thereby inducing inflammatory states in p16-3MR cells [60]. Other pharmaceuticals within this category, such as etoposide and camptothecin, exhibit similar senescence-inducing properties to doxorubicin [6]. Research indicates that low-dose etoposide (2 µM) can induce senescence in HepG2 liver cancer cells, while higher doses (100 µM) lead to apoptosis [62]. This effect may be attributed to increased activity of the checkpoint kinase Chk1, which is only observed in cells treated with low-dose etoposide. Chk1 phosphorylates p53, thereby modulating its activity [62]. Similarly, camptothecin has shown the ability to induce senescence only at lower doses [63]. Studies on the effects of camptothecin on various human colorectal cancer cell types (HCT116) found that only a lower dose (20 nM) induced senescence [63]. An important observation is that only cells capable of expressing p53 and p21 underwent senescence [63].

#### Alkylating agents

Alkylating agents represent a distinct category of anticancer drugs with a mechanism of action centered on the formation of an unstable alkyl group R-CH2+, which subsequently reacts with nucleophilic centers on proteins and nucleic acids. This intricate process leads to the inhibition of DNA replication and transcription [6]. Among these agents, busulfan stands out as a notable representative frequently employed in the treatment regimen for patients before allogeneic hematopoietic stem cell transplantation, particularly those diagnosed with chronic myeloid leukemia [64].

Research findings point to the capacity of busulfan to induce cellular senescence in various cell types, including mesenchymal stem cells, fibroblasts, and the cell lines of human osteosarcoma cells U2OS and MG63 [65–67]. It has been demonstrated that the mechanism of osteosarcoma senescence induction is based on the upregulation of microRNA-200, which subsequently downregulates the ZEB1 and ZEB2 genes [67]. Additionally, busulfan has been shown to accelerate the senescence process in murine bone marrow cells, thereby inhibiting their hematopoietic functions [68]. This constitutes a potential mechanism of bone marrow suppression observed in patients undergoing chemotherapy [68]. Another noteworthy drug exhibiting documented pro-senescence effects is temozolomide [60, 69]. Temozolomide induces senescence in both human and murine glioma cells and notably contributes to the accumulation of p16^INK4−^positive senescent cells in mice [60, 69]. The accumulation of these cells can induce a local or systemic inflammatory state, subsequently causing bone marrow suppression and heart dysfunction [60]. Over time, these cells may revert to proliferating cancer cells, resulting in disease recurrence [60].

#### Platinum-based compounds

Platinum-based compounds represent a crucial category of anticancer drugs extensively utilized in the treatment of solid tumors [70]. Through their interaction with DNA, these compounds induce the formation of both intra- and interstrand crosslinks, ultimately triggering apoptosis [71]. Pioneering this group is cisplatin, to exhibits proven pro-senescence effects [53]. Cisplatin alkylates and binds covalently the DNA, to form DNA cross-links [72]. This results in damage to the DNA strands and activation of the DDR [72]. DNA damage leads to the activation of p53/p21 or p16, resulting in the halting of proliferation and induction of senescence in cancer cells [60]. Cancer cells subjected to cisplatin therapy exhibited characteristic features of aging cells, such as flattened cell morphology, enlarged cell size, and increased expression of SA-β-gal [53]. Cisplatin displays its capability to induce cellular senescence in various types of cancer, including ovarian cancer, lung cancer, hepatocellular carcinoma, nasopharyngeal cancer, and specific melanoma cell lines [53, 73–76]. Advancements in drug development have led to the discovery of newer, better-tolerated platinum-based drugs with confirmed pro-aging effects [45, 77]. Carboplatin, for instance, demonstrates the ability to induce senescence in cancer cells obtained from patients with non-small cell lung cancer [45]. Similarly, oxaliplatin prompts the senescence in the same mechanism as cisplatin, of cancer cells extracted from rats with sizable tumors in the colon [45, 77]. In a study conducted by Cédric Seignez et al., an increased expression of SASP was observed in PROb colorectal cancer cells just 24 h after exposure to oxaliplatin treatment, which persisted for at least 9 days [77]. Using cytochemical detection with the X-Gal substrate and fluorescence detection with the DDAOG substrate, the activity of SA-β-Gal was demonstrated [77]. The team unequivocally confirmed oxaliplatin’s ability to induce the cellular aging process [77].

#### Aurora kinase inhibitors

Aurora kinase inhibitors are among the latest chemotherapeutic agents with promising potential [78]. By inhibiting Aurora-A, Aurora-B, and Aurora-C kinases, these compounds induce defects in mitotic spindle assembly, causing temporary cell cycle arrest and disrupting proper chromosome alignment during mitosis [78]. This disruption leads to polyploidy, failed cytokinesis, and endoreduplication [78]. Both mechanisms ultimately result in the death of cancer cells [78]. One example of an Aurora-A inhibitor in this class is AKI603 [79]. This drug can induce senescence in chronic myeloid leukemia (CML) cells [79]. Research shows that CML cells with the BCR-ABL-T315I mutation treated with AKI603 display features typical of senescent cells, such as increased SA-β-Gal activity, altered morphology, elevated levels of p53, p21, p27, and p16, as well as SASP secretion [79]. The exact mechanism underlying these effects is not yet fully understood, though researchers suggest a significant role for ROS accumulation caused by AURA-A inhibition [79]. Notably, researchers observed that the rise in p21 levels, a marker of senescence, was independent of p53, indicating that, unlike many previous drugs, AKI603-induced senescence may operate independently of p53 activity [79].

Another example of an Aurora kinase inhibitor capable of inducing senescence is MLN8054 [80]. This drug was shown to induce senescence both in vitro and in vivo in HCT116 models [80]. In both cases, cancer cells displayed morphological changes, increased SA-β-Gal activity, and elevated p53 and p21 levels following treatment [80].

#### CDK 4/6 inhibitors

Cyclin-dependent kinases, such as CDK4 and CDK6, are key regulators of the transition from the G1 to S phase in the cell cycle [81]. This makes them important targets in cancer therapy and gives them significant potential in inducing senescence [81]. One of the most extensively studied CDK4/6 inhibitors in the context of senescence induction is palbociclib [82–86]. This drug has demonstrated pro-senescence effects in various cancer types, including breast cancer, melanoma [82], gastric cancer [83, 84], glioma [85], and liposarcoma [86]. Research findings suggest that the mechanism of senescence induction by palbociclib is complex. Studies in breast cancer and melanoma cells show that the inhibition of FOXM1 expression by palbociclib plays an essential role in triggering senescence [82]. Meanwhile, in gastric cancer cells, palbociclib induced senescence not only through upregulation of p16, p21, and p53 but also by inhibiting the Notch pathway [84]. Researchers also highlight palbociclib’s unique ability to degrade the MDM2 gene in liposarcoma cells [86]. According to one study, only cells that had lost MDM2 gene functionality underwent senescence [86]. Abemaciclib is another CDK4/6 inhibitor capable of inducing senescence [87]. Research by Torres-Guzmán et al. indicates that abemaciclib can induce senescence in hormone receptor-positive (HR+) breast cancer cells [87]. This effect is highly dose-dependent, with only cells treated at lower doses undergoing senescence [87]. The pro-senescence mechanism of abemaciclib may involve FOXM1 inhibition, as suggested by research findings [87]. The third clinically used CDK4/6 inhibitor is ribociclib, which also shows potential pro-senescence effects [88]. Iyengar et al., in their study on the impact of ribociclib on serous ovarian cancer cells, demonstrated that ribociclib can induce a “pseudo-senescent” state in these cancer cells [88]. This term was used because the ribociclib-treated cancer cells exhibited typical senescence markers, such as elevated SA-β-Gal activity and certain SASP proteins, while still retaining the ability to proliferate [88].

### The impact of radiotherapy on inducing cellular aging

**Fig. 2:**
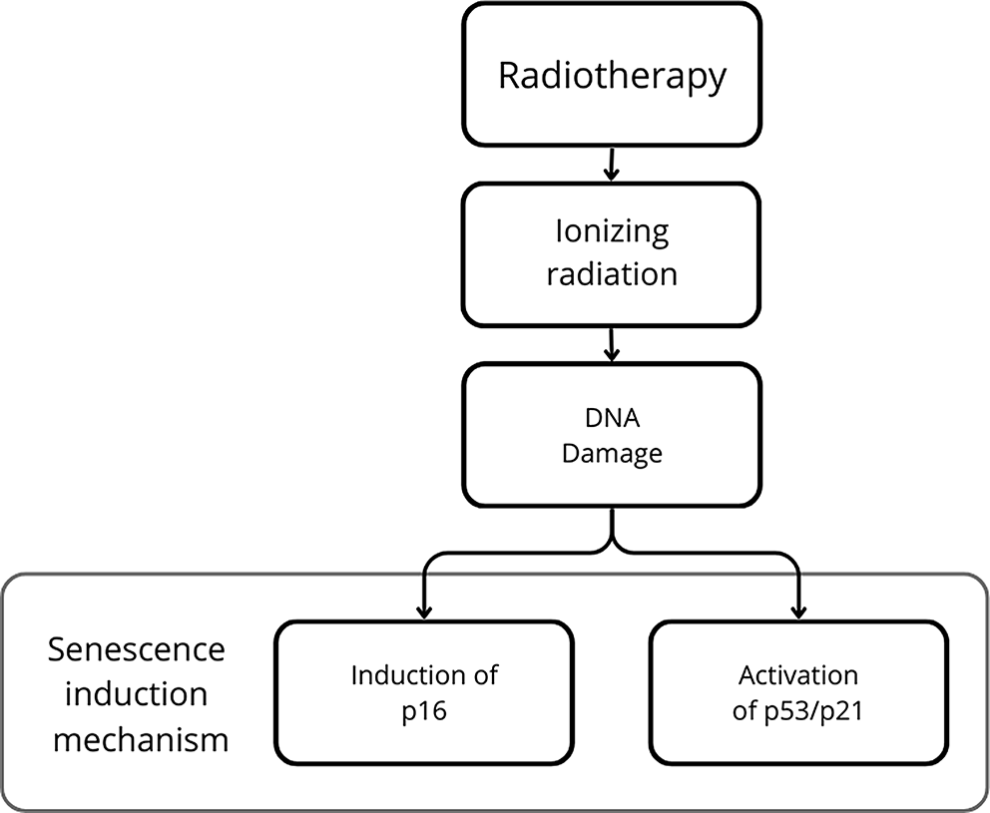
Presentation of the mechanisms of senescence induction by radiotherapy. Used in radiotherapy, ionizing radiation can induce senescence by causing DNA damage, activating the p53/p21 pathway, and inducing the expression of p16

Radiation therapy is a cancer treatment method known for its high effectiveness and lower risk of tissue damage compared to chemotherapy [52]. It is applied in the treatment of a wide range of tumors, most commonly in individual or combined therapies targeting cancers such as skin, breast, head and neck, lung, prostate, and bladder cancers [89]. Ionizing radiation (IR) is employed during radiotherapy, consisting of highly energetic electrically charged ions [89]. This leads to the formation of secondary charged particles and free radicals within the cancer cell, subsequently interacting with DNA [89]. As a result, extensive DNA damage occurs, leading to the death of cancer cells [89]. In contrast to chemotherapy, which is administered systemically, radiotherapy is applied locally, significantly reducing the likelihood of damage to healthy tissues [90] (Fig. 2).

We have evidence that radiotherapy, like chemotherapy, under certain conditions, does not lead to the immediate death of cancer cells but rather induces the process of senescence [91]. According to studies conducted by Jiang Ji and others, ionizing radiation affects primary keloid fibroblasts (KFb), causing damage to their DNA [90]. As a result, there is an overexpression of p16 and p21 [90]. These proteins, acting as cyclin-dependent kinase (Cdk) inhibitors, lead to the arrest of the G1 phase, and activation of the senescence pathway [90]. Moreover, markers of senescence, such as SA-β-gal and SASP, have been detected in patients treated with IR [91]. Other studies demonstrate the induction of SA-β-galactosidase due to the interaction of IR with breast cancer cell lines, colorectal cancer, immature neuroblastoma, and fibrosarcoma [92–94]. There are also studies indicating the significant impact of p53 status on the effect of IR on cells [94, 95]. According to studies conducted by K. R. Jones et al., breast cancer cells with missense mutations in the DNA-binding domain of p53 undergo apoptosis instead of senescence [94]. Approximately 80% of p53wild-type MCF-7 breast cancer cells exhibited signs of senescence (positive staining for b-galactosidase) as early as 7 days after exposure to a dose of 10 Gy ionizing radiation, concurrently demonstrating a minimal apoptosis rate [94]. Conversely, p53-mutant MDA-MB231 breast tumor cells showed a high apoptosis rate exceeding 30%, along with a low percentage of cells undergoing senescence [94]. Another study revealed that wild-type p53 in glioblastoma multiforme cells promoted the induction of senescence as an effect of IR treatment [95]. However, the same type of cells, but with mutations within p53, regained the ability to proliferate after IR treatment [95].

### Pro-senescence therapy

The concept of anticancer therapy where the induction of senescence is the main therapeutic goal has been accompanying us for over a dozen years [8]. Since cellular senescence is considered a natural defense mechanism against tumor development, and senescence inductions triggered by currently applied therapies can significantly impact their effectiveness, it is assumed that purposeful stimulation of senescence in cancer cells may be a promising direction in the development of anticancer therapy [8, 15, 55].

Inhibitors of CDK or those increasing the levels of CDK-inhibiting proteins constitute an intriguing group of pro-senescence compounds that can be utilized in anticancer therapy. Cancer cells are characterized by an elevated level of CDK, enabling them to progress through the cell cycle [96]. The use of CDK-inhibiting drugs, especially CDK4 and CDK6, induces cellular senescence by inhibiting the transition of the cell from the G1 to the S phase, simultaneously inhibiting its proliferation [81]. Currently, three CDK4 and CDK6 inhibitors approved by the FDA for the treatment of advanced breast cancer are available in therapy—palbociclib, abemaciclib and ribociclib [56]. In summary, while chemotherapy-induced senescence can be a significant aspect of cancer treatment, regulatory approval for these drugs is primarily contingent on their ability to effectively suppress tumor growth and improve clinical outcomes [56].

## Senotherapy

Senotherapy is an experimental treatment method aimed at eliminating the threat arising from the accumulation of senescent cells [97]. Senotherapeutic drugs can be divided into two main groups: senolytics and senomorphics [97]. Senolytics focus on eliminating senescent cells, while senomorphics prevent the harmful extrinsic effects of senescent cells by selectively targeting and inhibiting the development of the SASP [98]. Recent studies also highlight new groups of senotherapeutics. These include senolytics, which aim to block the transition of cells into a senescent state, and senoreversers, which promote the exit of senescent cells from this state [99]. Initially, research on senotherapeutics focused on age-related diseases [98]. It has been proven that the accumulation of senescent cells is responsible for tissue aging, and it has been observed that the pace of this process increases with age [100]. Therefore, compounds inhibiting the accumulation of senescent cells have become the subject of intensive research, especially for their potential application in treating diseases such as ischemic heart disease, Alzheimer’s disease, diabetes, atherosclerosis, or degenerative joint disease [98]. Increasing research indicating the adverse effects of senescent cancer cells on therapy prompted researchers to explore new therapeutic paths based on senotherapeutic drugs. As a result of these efforts, we have gained insights into the potential of senotherapeutics to induce the demise of senescent cells, thereby exerting anti-cancer effects. This breakthrough not only enhances treatment efficacy but also holds promise in overcoming drug resistance [97].

### Senolytics

Senolytics are a group of senotherapeutic drugs with confirmed effects in eliminating senescent cells [98]. Their action is based on various mechanisms but always leads to the induction of senescent cell death, consequently reducing their total number in the tissue [98]. Senolytics can be categorized into BCL family inhibitors, PI3K/AKT inhibitors, FOXO regulators, and other compounds [101].

#### BCL family inhibitors

The BCL family consists of proteins such as BCL-2, BCL-XL, and MCL-1, serving both proapoptotic and pro-survival functions [101]. Drugs inhibiting their action can lead to the removal of senescent cells [101].

The most researched drug in this group is Navitoclax (ABT-263). It acts by binding to BCL-W, BCL-2, and BCL-XL, inhibiting their function [102]. This mechanism impedes the binding and neutralization of pro-apoptotic proteins, Bax and Bak by BCL proteins [102]. Consequently, it leads to the disturbance of the outer mitochondrial membrane integrity, culminating in the induction of apoptosis [102]. Studies have shown its effectiveness in reducing the survival of senescent lung fibroblasts and umbilical vein endothelial cells [102]. Additionally, Navitoclax has been proven beneficial as a complementary therapy to conventional chemotherapy [72, 103, 104]. Research conducted by Marco Demaria et al. revealed that a seven-day Navitoclax therapy resulted in the elimination of senescent p16-3MR mouse cells induced by doxorubicin and contributed to delaying tumor recurrence and metastasis [60]. Furthermore, researchers noted that the use of combination therapy, consisting of doxorubicin and Navitoclax (responsible for removing aging cells), led to only a 20% decrease in the running activity of p16-3MR mice, compared to the therapy with doxorubicin alone, where this coefficient reached 50% [60]. Christin Tse et al., in their studies, demonstrated the high effectiveness of combining Navitoclax with Rituximab [103]. Both drugs were examined in the DoHH2 B-cell lymphoma flank xenograft model [103]. Neither of the drugs resulted in a permanent regression of the tumor; however, the combination of both drugs achieved a 70% complete tumor response and a 10% partial response [103]. Unfortunately, Navitoclax is not without flaws. Despite its effectiveness in eliminating senescent cells, it causes serious side effects such as thrombocytopenia and neutropenia [104]. Navitoclax’s precursor, ABT737, a BH3 mimetic, prevents the interaction between anti-apoptotic and pro-apoptotic proteins, also leading to senescent cell apoptosis [105]. Importantly, ABT-737 demonstrates proven senolytic properties against cancer cells. Studies indicate that ABT-737 effectively induces apoptosis and inhibits senescence in glioblastoma multiforme cells [106]. Administered at a dose of 2.5 µM, the drug had minimal effects on proliferating glioblastoma cells (LN229 and A172) while demonstrating significant senolytic properties against the same cell lines subjected to senescence induction by temozolomide [106]. The viability of senescent glioblastoma cells decreased by over 60% following a two-day treatment with ABT-737 (2.5 µM), whereas the viability reduction did not exceed 15% for proliferating cells [106]. However, unlike Navitoclax, which can be administered orally and has good bioavailability [102], ABT737 poorly dissolves in water, and its biological availability is low [87]. Among other derivatives of Navitoclax with proven senolytic effects, we can find compounds A-1155463 and A-1331852 [107, 108]. The senolytic properties of the compound A-1155463 have been demonstrated both in vitro and in vivo, particularly against non-small cell lung cancer (A549) cells induced into senescence by CCC-021-TPP [107]. Research findings indicate that administration of A-1155463 led to an increase in apoptosis among senescent cells while inhibiting tumor growth by more than 2.5 times compared to proliferating tumors and nearly twice as much compared to tumors treated only with the senescence inducer CCC-021-TPP [107]. The number of studies highlighting the senolytic effects of A-1331852 on cancer cells is limited. However, one study demonstrates the high efficacy of A-1331852 in eliminating senescent melanoma cells [108]. According to the findings, SK-MEL-103 and SK-MEL-28 cells induced into senescence by IR or palbociclib showed approximately 40% higher mortality following A-1331852 treatment compared to cells subjected only to chemotherapy or radiotherapy [108].

Panobinostat is a drug registered in the EU since 2015 for the treatment of relapsed/refractory multiple myeloma [109]. Its ability to combat tumors is attributed to the inhibition of histone deacetylase [109]. Studies from 2017 showed that panobinostat is also capable of effectively removing senescent cancer cells accumulated during chemotherapy, through the inhibition of BCL-XL [110]. Panobinostat effectively eliminated senescent cells in both non-small cell lung cancer (NSCLC) and head and neck squamous cell carcinoma (HNSCC) [110].

#### PI3K/AKT inhibitors

Activation of phosphoinositide 3-kinase (PI3K) can lead to the phosphorylation and subsequent inactivation of Bad and caspase-9 [111]. This action protects the cell from programmed death, simultaneously safeguarding senescent cells from apoptosis [111]. It can be inferred that inhibiting this process may contribute to the elimination of senescent cancer cells.

The first senolytic drugs discovered based on hypotheses were dasatinib and quercetin [112, 113]. Dasatinib is a classical anticancer drug, a tyrosine kinase inhibitor, which also exhibits senolytic effects [114]. Quercetin is a well-known natural flavonoid that, by inhibiting the activity of mTOR and PI3K, demonstrates senolytic properties [114]. This phenomenon occurs because the Pi3K/mTOR pathway constitutes one of the key intracellular signaling pathways [115]. The significance of this pathway appears to increase in the case of cancer cells, where it is responsible for stimulating cell growth and proliferation [115]. Numerous studies demonstrate the satisfactory senolytic effects of combined therapy with dasatinib and quercetin (D + Q) on non-cancerous cells [97]. However, there is a lack of studies confirming the efficacy of this combination in eliminating senescent cancer cells. Research conducted by Kovačovicova et al. demonstrated that D + Q was unable to eliminate senescent hepatocellular carcinoma (HCC) cells that had entered a state of senescence following prior doxorubicin treatment [116]. Furthermore, the researchers showed that treatment of HCC with D + Q alone could even contribute to tumor progression [116]. This does not imply, however, that the senolytic properties of D + Q are irrelevant in cancer therapy. As Wang et al. indicate, the ability of D + Q to eliminate senescent adipose-derived stem cells (ADSCs) could be beneficial in limiting ovarian cancer metastasis to the peritoneum and adipose tissue [117]. According to their findings, ADSCs induced into senescence by carboplatin or olaparib (a drug used in ovarian cancer therapy) promoted the development and migration of ovarian cancer cells [117]. Inhibiting this process through the elimination of senescent ADSCs by D + Q significantly reduced ovarian cancer metastasis to adipose tissue [117]. In addition to quercetin, there are two other flavonoids with potential senolytic effects – myricetin and fisetin [118, 119]. Myricetin can counteract photoaging and platelet aggregation, as well as demonstrate anticancer properties [118]. Researchconducted by Ye Li et al. indicates that myricetin therapy reduced the number of adenomatous polyps by 58.9% in the small intestine and 71.8% in the colon (compared to the carrier control) in APC Min/+ mice characterized by spontaneous tumorigenesis in these segments of the intestine [120].

Fisetin is another natural flavonoid in this group of drugs, commonly found in many fruits and vegetables such as apples, onions, or strawberries [121]. It is considered a potent antioxidant with proven efficacy in reducing the risk of vascular diseases and ischemic heart disease [121, 122]. Fisetin is also a compound with senolytic properties [119]. Like previously mentioned flavonoids, it can disrupt the PI3K/AKT pathway, leading to the elimination of senescent cells [119]. In their research, Maria Russo et al. demonstrated that fisetin effectively eliminates senescent cells in radio-resistant colorectal cancer (HT500) [123]. In vitro studies showed that fisetin could eradicate over 60% of senescent HT500 cells [123]. The researchers proposed that the senolytic effect observed in these cells is attributable not only to the disruption of the PI3K/AKT pathway but also to interference with the ERK/MEP pathway, which plays a crucial role in regulating inflammation and resistance to apoptosis [123].

Some PI3K/AKT inhibitors, such as fisetin, are considered to be safe compounds with a minimal risk of adverse effects [124]. However, the majority of them may still induce certain undesirable effects, including stomatitis, hyperlipidemia, rash, myelosuppression, and hyperglycemia [125]. Additionally, women undergoing breast cancer treatment may experience fatigue, anorexia, headache, and diarrhea [125].

#### FOXO regulators

FOXO transcription factors play a crucial role in the human body [126]. They participate in the regulation of survival, growth, and oxidative stress management of cells, and also influence DNA repair processes and apoptosis [126]. Additionally, FOXO can counteract the elimination of senescent cells by interacting with p53 and inhibiting apoptosis [127]. As a result of these properties, the FOXO4-DRI peptide was designed. Thanks to its higher affinity for the p53 domain, this peptide disrupts the interaction between natural FOXO factors and p53, leading to the induction of apoptosis in senescent cells [127]. According to studies conducted by Marjolein P. Baar et al., FOXO4-DRI reduced the lifespan of primary human fibroblasts IMR90 induced into senescence by ionizing radiation by over 11 times (11.73 times), compared to the control group consisting of non-senescent IMR90 cells [127]. This suggests a high effectiveness and significant selectivity of FOXO4-DRI towards senescent cells [127]. The impact of FOXO4-DRI on p16::3MR mice, which experienced cellular senescence induction after doxorubicin administration, was investigated [127]. FOXO4-DRI effectively counteracted doxorubicin-induced cell senescence and additionally mitigated doxorubicin-induced loss of body mass and elevated levels of aspartate aminotransferase (AST), contributing to the reduction of the hepatotoxicity of the chemotherapy agent [127]. Currently, no studies suggest that FOXO4-DRI has the capacity to eliminate senescent cancer cells. As such, its application may be limited to a supportive role in cancer therapy, primarily focused on alleviating chemotherapy-associated side effects.

A different approach was presented by researchers led by Hillary H. Le et al. [128]. Their research focused on discovering senolytic peptides with a higher affinity for FOXO4 than for p53 [128]. Among the peptides identified, the one with the highest therapeutic potential was named ES2 [128]. The senolytic properties of ES2 were initially demonstrated in an in vitro study, where its effects were tested on both proliferating and senescent A375 melanoma cells (senescence was induced by doxorubicin treatment) [128]. The tested peptide led to a more than threefold decrease in the viability of senescent cells compared to proliferating cells, with an IC50 concentration (for senescent cells) of 8 µM [128]. The effectiveness of the ES2 peptide was further confirmed in in-vivo studies [128]. In this study, mice were injected into the ears with either 750,000 or 25,000 human A375 melanoma cells, followed by a 2-day treatment, starting 24 h later, with either saline (control group), ES2 peptide, dabrafenib (a senescence inducer), or a combination of ES2 and dabrafenib [128]. Mice were euthanized when the tumor reached 2 cm in size [128]. For mice injected with a larger number of tumor cells, survival in the monotherapy groups was comparable to that of the control mice [128]. However, the combination therapy resulted in a 50% increase in survival time [128]. Among mice injected with a smaller number of tumor cells, the efficacy of the combination therapy was even greater, with a 65% increase in survival time [128]. In both in vitro and in vivo studies, the ES2 peptide has shown potent senolytic activity with minimal apoptotic effects on non-senescent cancer cells [128]. Researchers attribute ES2’s effectiveness to its ability to bind to FOXO4, thereby disrupting the interaction between FOXO4 and p53 [128].

As FOXO proteins constitute key components of the insulin signaling pathway, the impact of drugs regulating its action significantly influences blood sugar levels [115]. Consequently, their usage in individuals with impaired glucose metabolism, such as those with diabetes, should be restricted or closely monitored [115] (Table 1).

**Table 1:** Selected senolytic drugs and their effects on cancer treatment

Senolytic molecule	Mechanism of action	Effect	References
*Effect on cancer cells*			
Navitoclax	Inhibition of BCL-2, BCL-XL and MCL-1	Reduction of senescent lymphoma cells	[103]
ABT 737	BH3 mimetic	Induction of apoptosis and reductrion of senescent glioblastoma multiforme cells	[106]
A-1155463	Inhibition of BCL-XL	Reduction of non-small lung cancer A549 cells and inhibition of tumor growth	[107]
A-1331852	Inhibition of BCL-XL	Reduction of melanoma cells induced by IR	[108]
Panobinostat	Inhibition of BCL-XL	Reduction of NSCLC and HNSCC cells	[110]
Dasatinib + Quercetin	Inhibition of the activity of mTOR and PI3K	Lack of effectiveness in eliminating senescent cancer cells	[116]
Myricetin	Inhibition of the activity of mTOR and PI3K	Reduction number of adenomatous polyps in mice small intestine and colon	[120]
Fisetin	Inhibition of the activity of mTOR and PI3K	Reduction of senescent radio-resistant colorectal cancer HT500 cells	[123]
ES2	Disrupts the interaction between natural FOXO factors and p53	Reduction of senescent human melanoma A375 cells induced by doxorubicin	[128]
		Reduction of senescent melanoma tumor growth induced by dabrafenib	[128]
*Effects on non-cancerous cells*			
Navitoclax	Inhibition of BCL-2, BCL-XL and MCL-1	Reduction of senescent p16-3MRmouse cells induced by doxorubicin, and delayed tumor recurrence and metastasis	[60]
Dasatinib + Quercetin	Inhibition of the activity of mTOR and PI3K	Reduction of senescent ADSCs - reduction of ovarian cancer metastases	[117]
FOXO4-DRI	Disrupts the interaction between natural FOXO factors and p53	Reduction of doxorubicin-induced senescent cancer cells – reduction of side effects	[127]

### Senomorphics

Senomorphic drugs constitute another group of senotherapeutic drugs [97]. Unlike senolytics, they do not reduce the number of already existing senescent cells [98]. The action of senomorphic drugs is based on delaying or preventing cell aging by controlling or reducing SASP [98].

Nordihydroguaiaretic acid (NDGA) exhibits proven senomorphic properties, extending life by 8–10% in male mice [129]. NDGA acts by inactivating lipoxygenase, increasing the catabolism of fatty acids [130]. Additionally, it increases the expression of the peroxisome proliferator-activated receptor α (PPARα) and phosphorylated form of AMP-activated protein kinase, improving the regulation of dyslipidemia and increasing lipid metabolism efficiency [130].

Spermidine is a polyamine with proven anti-aging effects [131]. Recent studies have shown that high doses of this substance led to a reduction in the acetylation of many lysine residues located on the N-terminal tail of histone H3 [131]. This occurs due to the ability of spermidine to inhibit histone acetyltransferases [131]. This action subsequently leads to changes in chromatin structure, activation of gene transcription, and may stimulate antioxidant mechanisms and cellular protective responses [131]. This could result in the cessation of cell senescence, extension of its lifespan, and induction of autophagy [131].

Other drugs with potential anti-senescence effects are fluvastatin and valsartan, traditional medications used in cardiovascular diseases [132]. Research conducted by Miodrag Janić et al. indicates that the combination of both drugs, administered in low doses for 30 days to middle-aged men with impaired functional and structural characteristics of arterial walls, leads to an increase in the expression of Sirt1 (1.8 times), telomerase activity, the catalytic subunit of AMP α 2 protein kinase (PRKAA) (1.5 times), and the KLOTHO gene (1.7 times) after just 30 days of treatment [132]. Additionally, they lead to the activation of several intracellular protective pathways [132–134]. Increasing the expression of the PRKAA gene leads to the upregulation of the catalytic subunit of AMPK, which serves as the main regulator of cellular response and is responsible for maintaining energy balance within the cell [132, 133]. This suggests that the use of these drugs may contribute to cell protection [132, 133]. KLOTHO functions as a co-receptor in several crucial intracellular signaling pathways, and its reduced expression in the body may result in cardiovascular diseases, induction of inflammation, or promotion of aging [134]. Therefore, the increased expression of KLOTHO resulting from treatment with valsartan and fluvastatin may yield highly positive effects in inhibiting cell senescence and promoting overall cellular protection [132, 134] (Table 2).

**Table 2:** Selected senomorphic drugs and their effects

Senomorphic molecule	Mechanism of action	Effect	References
Nordihydroguaiaretic acid (NDGA)	Inactivating lipoxygenase Increasing the expression of PPARα	Improving the regulation of dyslipidemia and increasing lipid metabolism efficiency Extending life by 8–10% in mice	[129, 130]
Spermidine	Inhibition of histone acetyltransferases	Stimulation of antioxidant mechanisms and cellular protective responses	[131]
Fluvastatin + Valsartan	Increasing the expression of the PRKAA and KLOTHO gene	Activation of several intracellular protective pathways Improving the energy balance within the cell	[132–134]

## One-Two Punch therapy

**Fig. 3:**
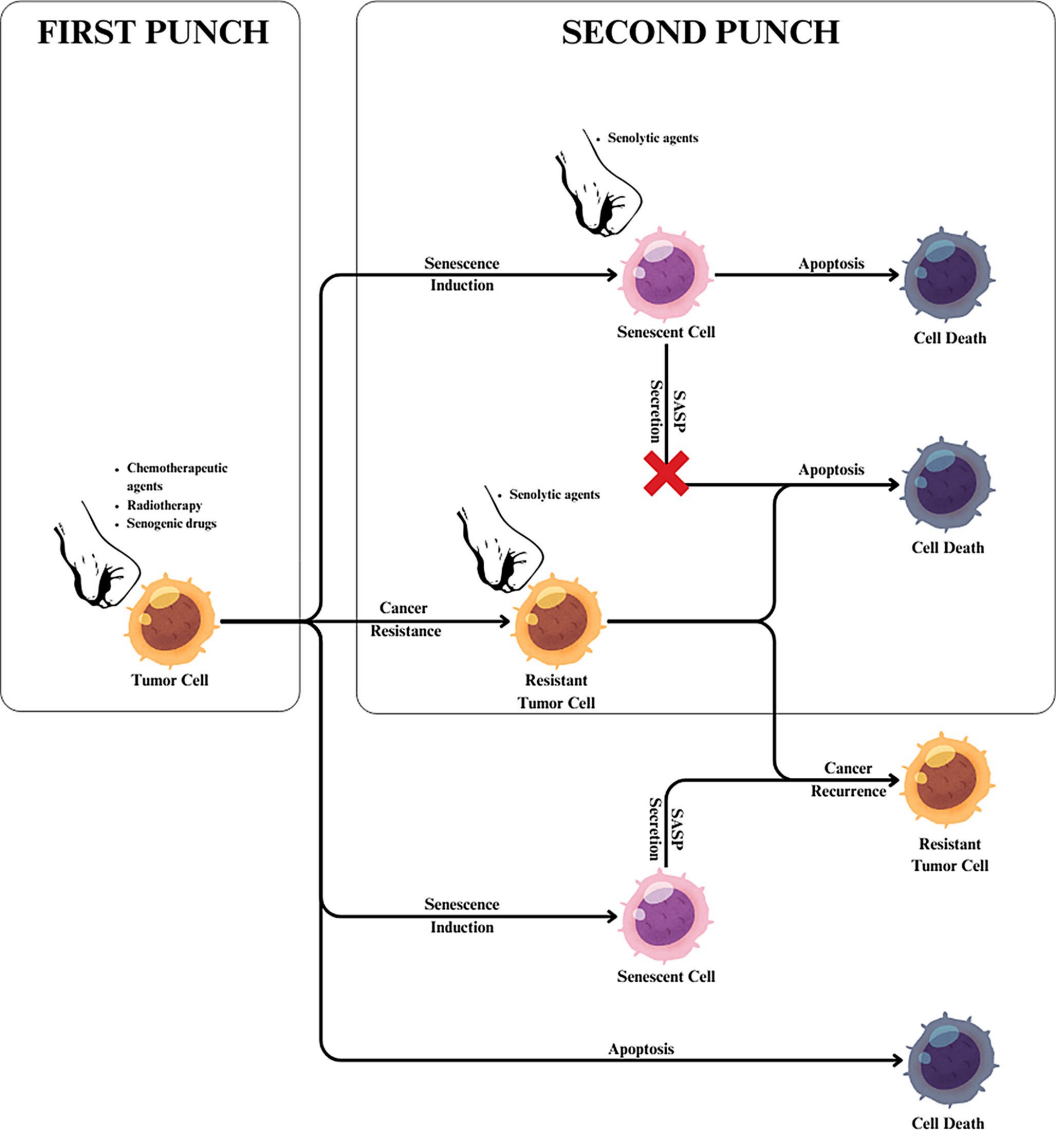
Presentation of the main stages of One-Two Punch therapy

Currently employed anticancer therapies, such as chemotherapy or radiotherapy, exhibit proven senescence-inducing effects [54]. In certain conditions, this effect is considered beneficial as it can impede tumor growth, delay disease progression, and even lead to the elimination of some cancer cells through the induction of an immune response [55]. However, an increasing body of research indicates the negative impact of accumulating senescent cancer cells, whose aggregation may result in therapy failure and even disease relapses [6]. This knowledge has paved the way for new applications of senolytic drugs and the development of an innovative treatment method called “One-Two Punch” Therapy [72]. The “One-Two Punch” Therapy involves the use of a chemotherapeutic drug with proven senescence-inducing effects initially, followed by the administration of a senolytic drug [72]. The use of a senescence-inducing drug leads to the induction of senescence in cancer cells, making them more susceptible to the action of senolytics, leading to their elimination [72] (Fig. 3).

### Possible strategies of “One-Two Punch” therapy

The inaugural investigations delving into the potential of this therapeutic strategy surfaced in the year 2017 [135]. A549 and H358 KRAS mutant lung cancer cells examined in vitro were treated with potent senescence inducers, such as aurora kinase inhibitors: Alisertib and Barasertib [135]. As a result of the action of these substances, the cells became more susceptible to BCL-2 family inhibitors, especially the compound ABT-263, which has proven senolytic properties [135].

Research conducted by Cun Wang’s team demonstrated the effective utilization of the senogenic compound (also known as a senescence inducer) XL413 and the senolytic compound AZD8055 in liver cancer therapy [136]. XL413, an inhibitor of CDC7 kinase, effectively induced senescence in liver cancer cells, simultaneously sensitizing them to the senolytic action of the mTOR inhibitor, AZD8055 [136]. Mice with hepatocellular carcinoma and treated with XL413 (100 mg/kg) in combination with AZD8055 (20 mg/kg) exhibited significantly greater tumor mass reduction and increased lifespan compared to mice treated with monotherapy using either of these drugs [136]. Furthermore, in mice treated with the combination of these drugs, there was a noteworthy decrease in SA-β-gal positive cells and p16 (INK4A) positive cells, indicating that AZD8055 effectively eliminated aging cells [136].

Satisfactory therapeutic effects were also observed with the combined use of olaparib and ABT-263 [137]. Olaparib, an inhibitor of poly(ADP-ribose) polymerase 1 (PARP), demonstrated proven senogenic effects on ovarian cancer cells [137]. The utilization of olaparib, as well as other PARP inhibitors, in combination with ABT-263, showed high efficacy in eliminating cancer cells [137]. Among the 4 high-grade serous epithelial ovarian cancer (HGSOC) cell lines: OV1369(R2), OV90, OV4453, and OV1946, the percentage of dead tumor cells after treatment with olaparib (10µM) and ABT-263 (0.25 µM for OV1369(R2) and 2.5 µM for other cell lines) exceeded 75% [137]. This index was significantly lower, not exceeding 30%, in the case of monotherapy, except for the OV1946 cell line, where the percentage was approximately 60% for ABT-263 and about 50% for olaparib [137].

One of the well-known senolytic drugs, Navitoclax, also holds the potential for use in “One-Two Punch” therapy [138]. As a BCL-2 inhibitor, Navitoclax neutralizes the drug resistance of senogenic BRAF inhibitor, vemurafenib [138]. This broadens the applicability of vemurafenib to a wider spectrum of tumors, offering a promising treatment option for cancers harboring the BRAFV600E mutation, such as papillary thyroid carcinoma (PTC), melanoma, or non-small cell lung cancer [138].

The therapeutic potential is also demonstrated by the combination of fisetin and sorafenib [139]. These drugs, through caspase-3 and caspase-8, synergistically induced apoptosis in HeLa cells [139]. Therapeutic outcomes in the treatment of cervical cancer were found to surpass the results achieved by these drugs individually [139]. The administration of fisetin (40 µM) and sorafenib (5 µM) for 24 h significantly reduced the viability of human cervical cancer HeLa cells, reaching approximately 20% compared to the control group [139]. Moreover, it contributed to the induction of apoptosis in about 50% of the cells [139]. In monotherapy, the most promising results were observed with the administration of 40 µM fisetin, leading to a reduction in the viability of cancer cells to approximately 50% compared to the control group and the induction of apoptosis in about 20% of the cells [139].

Digoxin is a cardiac glycoside that, due to its senolytic action, possesses limited anticancer effects [140]. However, in combination with the classical chemotherapy agent with similarly limited anticancer effects, gemcitabine, the effectiveness of the therapy significantly increases [140]. Francisco Triana-Martínez et al., in their research, administered gemcitabine alone (25 mg/kg, IP), digoxin alone (2 mg/kg, IP), or a combination of both drugs at the same doses through intraperitoneal injections to immunocompromised mice that had previously been subcutaneously injected with A549 lung adenocarcinoma cells [140]. The application of both drugs contributed to a considerable reduction in tumor volume in mice, decreasing from 100 mm3 to less than 3 mm3 after 21 days [139]. Furthermore, it even led to the disappearance of portions of the tumors following a 3-week therapy [140]. In mice subjected to monotherapy, the tumor volume slightly increased over the same period [140].

### Limitations of “One-Two Punch” therapy

“One-Two Punch” therapy, like any other form of treatment, faces certain limitations and challenges. An ideal scenario would involve the use of pro-senescence drugs with high efficacy in inducing senescence in a substantial proportion of cancer cells [60]. Unfortunately, currently known compounds, even if characterized by high effectiveness, often act on a limited number of cancer cell types [60]. Moreover, their action frequently extends beyond cancer cells, affecting healthy cells and causing harmful side effects [50]. Another issue is the difficulty in determining the specific moment at which cancer cells undergo senescence [5]. To achieve the best results of senolytic drug therapy, it is essential to identify the appropriate timing of drug administration. Currently, there is no unified standard for senescence biomarkers [16]. The levels of many of these markers may vary depending on the type of cancer cell undergoing senescence or the cause of senescence induction [16]. Developing broad-spectrum senolytic drugs poses yet another challenge [5]. Similar to senogenic drugs, it would be advantageous to create senolytics effective in eliminating a wide range of senescent cells [5]. Unfortunately, current senolytics exhibit high variability in efficacy, depending on the type of cell undergoing senescence [141].

The last challenge described here involves a more precise determination of the impact that senescent cells exert on their microenvironment and the entire human body [5]. This is particularly significant in the context of wound healing, where senescent cells play a supportive role in regenerative processes. Senescent fibroblasts and endothelial cells, through the secretion of SASP—particularly PDGF-A—promote an increase in myofibroblast numbers at wound sites, which positively affects wound healing [141]. However, the use of senolytics, which eliminate these senescent cells, or senomorphics, which modify SASP secretion, could impair wound healing, including that associated with tumor-related wounds. Senotherapeutics may also accelerate liver fibrosis, a particularly significant concern for patients with liver injuries [142]. This occurs because the senescence of hepatic stellate cells, which are partly responsible for forming fibrotic scar tissue, acts as a natural mechanism that delays and limits fibrotic changes in response to injury [142, 143].

## Concluding remarks

Cellular senescence plays a key role in cancer development, presenting both a significant challenge and a promising therapeutic target. The accumulation of senescent cells contributes to cancer progression and increased resistance to treatment, emphasizing the need for new therapeutic strategies. Senolytics, drugs that eliminate senescent cells, show great potential in oncology, offering the possibility of selectively targeting cancer cells that enter a state of senescence without affecting healthy cells. Approaches such as “One-Two Punch” therapy, which combines senescence-inducing agents with senolytics, can significantly enhance treatment efficacy, reduce the risk of recurrence and resistance, and pave the way for more precise, personalized cancer therapies.

While this study highlights the promising potential of senotherapy in oncology, several limitations warrant consideration. One of the primary challenges is the identification and standardization of appropriate biomarkers for senescence. The complexity of markers associated with cellular aging complicates the determination of optimal timing for administering senolytics, leading to inconsistencies in therapeutic outcomes and variable clinical efficacy.

Additionally, the diversity in cancer cell responses to senolytics presents another significant limitation. The effectiveness of available senolytics can vary widely depending on the type of cancer cells and the specific mechanisms by which they undergo senescence. This variability underscores the need for further research focused on developing broad-spectrum senolytics capable of targeting a wider range of cancer types effectively. However, because of the complexity and diversity of cellular senescence, creating such broadly effective senolytics may be very difficult or even impossible. A different and possibly better approach could be to develop senolytic therapies tailored to specific cancer types or senescence patterns. This personalized strategy, based on the principles of personalized medicine, could allow for more effective targeting of senescent cells in specific situations, offering another promising direction for advancing senotherapy.

Moreover, the interaction between senescent cells and their microenvironment is an area that requires deeper exploration. While senescent cells contribute to essential processes such as wound healing, their elimination may inadvertently hinder tissue regeneration and overall healing. Therefore, it is crucial to advance the development of more selective therapies that enable the removal of undesirable senescent cells while preserving beneficial regenerative processes within the body.

In summary, although this study presents senotherapy as a promising alternative for enhancing anticancer strategies, numerous aspects of its application necessitate further investigation. Addressing these limitations will be vital for refining therapeutic approaches and better tailoring interventions to meet patient needs.

## Data Availability

No datasets were generated or analysed during the current study.
